# Correction: Anthropological responses to environmental challenges in SAARC nations: A comparative analysis

**DOI:** 10.1371/journal.pone.0299863

**Published:** 2024-02-27

**Authors:** Chunyan Liu, Muneeb Ahmad, Ali Altalbe

The second author, Muneeb Ahmad, is incorrectly noted as the corresponding author. The correct corresponding author is Ali Altalbe. Ali Altalbe’s email address is: a.altalbe@psau.edu.sa.

The published funding statement is inaccurate. The correct funding statement is as follows: The authors extend their appreciation to Prince Sattam bin Abdulaziz University for funding this research work through the project number (PSAU/2024/01/224252).
